# Blood Co-Circulating Extracellular microRNAs and Immune Cell Subsets Associate with Type 1 Diabetes Severity

**DOI:** 10.3390/ijms21020477

**Published:** 2020-01-11

**Authors:** Silvia Garavelli, Sara Bruzzaniti, Elena Tagliabue, Francesco Prattichizzo, Dario Di Silvestre, Francesco Perna, Lucia La Sala, Antonio Ceriello, Enza Mozzillo, Valentina Fattorusso, Pierluigi Mauri, Annibale A. Puca, Adriana Franzese, Giuseppe Matarese, Mario Galgani, Paola de Candia

**Affiliations:** 1 IRCCS MultiMedica, 20138 Milan, Italy; silvia.garavelli@multimedica.it (S.G.); elena.tagliabue@multimedica.it (E.T.); francesco.prattichizzo@multimedica.it (F.P.); lucia.lasala@multimedica.it (L.L.S.); antonio.ceriello@multimedica.it (A.C.); annibale.puca@multimedica.it (A.A.P.); 2 Istituto per l’Endocrinologia e l’Oncologia Sperimentale “G. Salvatore”, Consiglio Nazionale delle Ricerche, 80131 Naples, Italy; 3 Dipartimento di Biologia, Università degli Studi di Napoli “Federico II”, 80126 Naples, Italy; 4 Istituto di Tecnologie Biomediche, Consiglio Nazionale delle Ricerche (ITB-CNR), 20090 Segrate (MI), Italy; dario.disilvestre@itb.cnr.it (D.D.S.); pierluigi.mauri@itb.cnr.it (P.M.); 5 Dipartimento di Medicina Clinica e Chirurgia, Università degli Studi di Napoli “Federico II”, 80131 Naples, Italy; francesco.perna@unina.it; 6 Centro Regionale di Diabetologia Pediatrica, Dipartimento di Scienze Mediche Traslazionali, Sezione di Pediatria, Università degli Studi di Napoli “Federico II”, 80131 Naples, Italy or mozzilloenza@gmail.com (E.M.); vfattorusso1@gmail.com (V.F.); franzese@unina.it (A.F.); 7 Dipartimento di Medicina, Chirurgia e Odontoiatria ”Scuola Medica Salernitana”, Università di Salerno, Via S. Allende, 84081 Baronissi (SA), Italy; 8 Dipartimento di Medicina Molecolare e Biotecnologie Mediche, Università degli Studi di Napoli “Federico II”, 80131 Naples, Italy

**Keywords:** autoimmune, lymphocytes, microRNAs, biomarkers

## Abstract

Immune cell subsets and microRNAs have been independently proposed as type 1 diabetes (T1D) diagnostic and/or prognostic biomarkers. Here, we aimed to analyze the relationships between peripheral blood circulating immune cell subsets, plasmatic microRNAs, and T1D. Blood samples were obtained from both children with T1D at diagnosis and age-sex matched healthy controls. Then, immunophenotype assessed by flow cytometry was coupled with the quantification of 60 plasmatic microRNAs by quantitative RT-PCR. The associations between immune cell frequency, plasmatic microRNAs, and the parameters of pancreatic loss, glycemic control, and diabetic ketoacidosis were assessed by logistic regression models and correlation analyses. We found that the increase in specific plasmatic microRNAs was associated with T1D disease onset (let-7c-5p, let-7d-5p, let-7f-5p, let-7i-5p, miR-146a-5p, miR-423-3p, and miR-423-5p), serum C-peptide concentration (miR-142-5p and miR-29c-3p), glycated hemoglobin (miR-26a-5p and miR-223-3p) and the presence of ketoacidosis (miR-29c-3p) more strongly than the evaluated immune cell subset frequency. Some of these plasmatic microRNAs were shown to positively correlate with numbers of blood circulating B lymphocytes (miR-142-5p) and CD4^+^CD45RO^+^ (miR-146a-5p and miR-223-3p) and CD4^+^CD25^+^ cells (miR-423-3p and miR-223-3p) in children with T1D but not in healthy controls, suggesting a disease-specific microRNA association with immune dysregulation in T1D. In conclusion, our results suggest that, while blood co-circulating extracellular microRNAs and immune cell subsets may be biologically linked, microRNAs may better provide powerful information about T1D onset and severity.

## 1. Introduction

Type 1 diabetes (T1D) is an autoimmune multi-factorial disease characterized by the destruction of insulin-producing β cells of the pancreatic islets which results in chronic inflammation and a progressively severe insulin deficit [1,2,3,4]. T1D is one of the most common chronic diseases among children, with an incidence that has increased in children younger than 15 years of age, and it is caused by the interplay of environmental, immunological, and genetic factors [5].

At T1D diagnosis, a high amount of β cell mass has already been destroyed, and the remaining cell mass progressively declines over time [1,6]. In particular, without proper clinical management for each patient, T1D progression leads to serious complications that reduce quality of life and life expectancy; care of and outcomes for these patients would instead greatly improve through an understanding of the mechanisms at the basis of disease complications.

A sub-group of patients has been shown to maintain an appreciable endogenous ability to produce insulin, as assessed through the monitoring of serum concentration of C-peptide, secreted by β cells at a one-to-one ratio with insulin. Even if typically low, this insulin production is very important since it has been clinically associated with longer periods of remission (i.e., a honeymoon phase) and less severe diabetes-related vascular complications [7,8]. Beside serum concentration of C-peptide, diabetic ketoacidosis (DKA) is most critical in the characterization of T1D. DKA is a serious, potentially lethal complication of T1D and may represent the first diagnostic sign for up to 40% of diabetic patients [9,10]. The presence of DKA in new onset T1D patients is associated with less residual β cell function and lower glycemic control, and places T1D subjects at an increased risk of poorer prognosis for morbidity and mortality [11,12].

Despite much effort, the immunopathogenesis of T1D, DKA, and other T1D-related complications still remains unclear, hence appropriate therapeutic targets and/or strategies to block or at least delay islet β cell failure and prevent the development of diabetic complications are still lacking. Many studies have described how T1D development is associated with changes in various peripheral blood circulating cell populations, and their asset might be associated with clinical and age-related differences in T1D patients [13,14,15,16]. It has been shown that in T1D subjects, the number of natural killer (NK), dendritic (DC), and T cell subsets is significantly altered compared to controls [17]. Moreover, the immunological profile significantly changes over time in T1D, and a specific peripheral immune cell signature can be associated with worsening disease [18].

In the last five years, together with immune cell subsets, several studies have proposed blood circulating microRNAs (miRNAs) as novel promising biomarkers for metabolic and autoimmune diabetes and related complications [19,20,21,22,23,24,25,26,27,28,29,30,31]. Inside cells, these small (~22 nucleotides in length) and highly conserved non-coding RNAs regulate mRNA decay and protein translation [32]. The biological influence of miRNAs is broad since the majority of mRNAs are miRNA conserved targets (miRBase.org), and, as a consequence, miRNAs have the capability to regulate highly diversified biological processes, including pathological mechanisms [33,34]. A more recent finding is that miRNAs are released in the extracellular space not only as byproducts of cell loss/damage but also as mediators of cell-to-cell communication at a paracrine level and among different tissues [35]. Outside the cells, their stability is ensured by their association with different protective cellular components such as protein complexes or extracellular vesicles [36,37,38,39]. On the one hand, dysregulation of blood circulating miRNAs can mirror a tissue-specific dysfunction; on the other, these extracellular miRNAs are able to orchestrate distant regulatory mechanisms. In other words, they may be used as both novel disease biomarkers and therapeutic targets [40,41].

Despite the relevance of both peripheral blood circulating immune cell subsets and extracellular miRNAs, scant information is available on whether these parameters are co-modulated in the onset of T1D and whether their linkage is associated with pancreatic loss, glycemic control, and DKA in children with T1D. To evaluate this interconnection and its potential clinical utility in discriminating disease onset and severity, we here enumerate 18 blood circulating immune cells, and, in parallel, profile 60 plasmatic miRNAs in T1D children at diagnosis and healthy controls.

## 2. Results

### 2.1. Plasmatic miRNAs Are Better Than Peripheral Blood Circulating Immune Cells in Discriminating Type 1 Diabetics from Healthy Children

**Figure 1:**
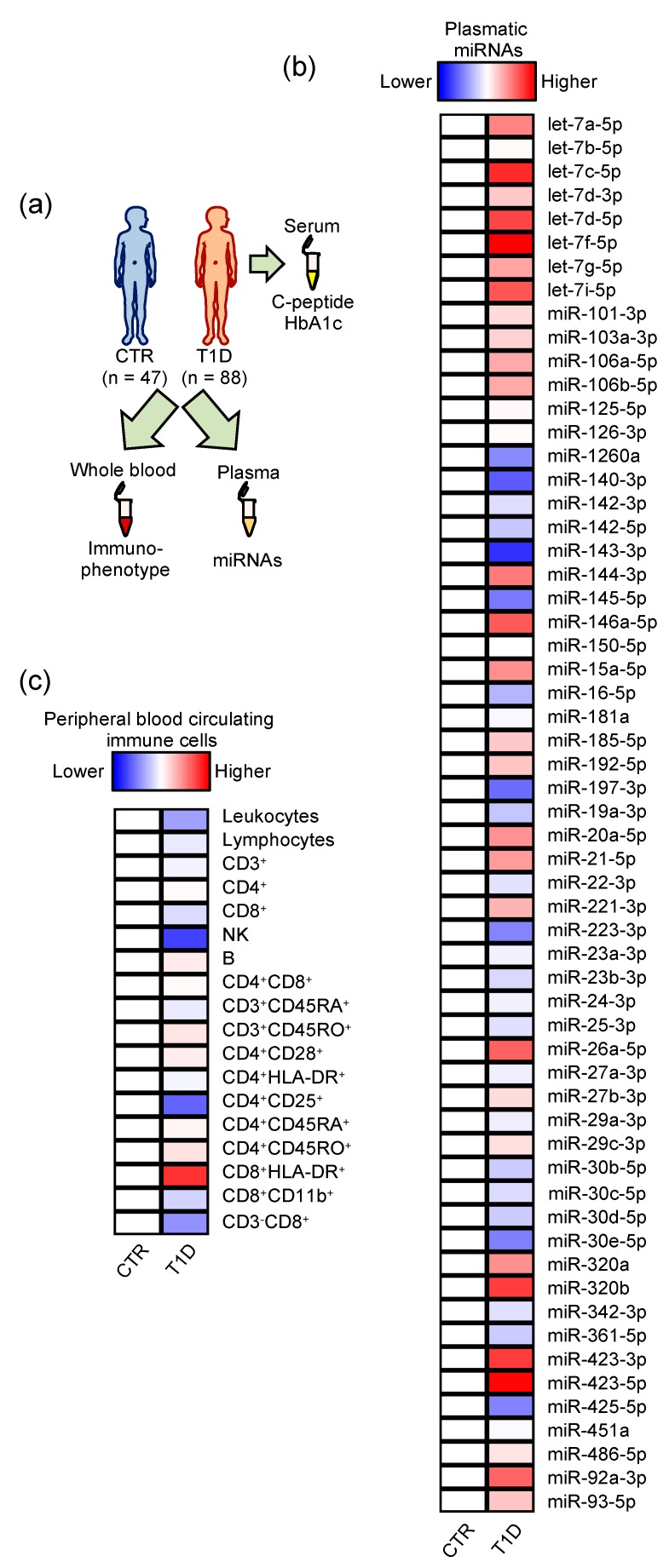
Schematic overview of the study. (**a**) Subjects recruited for the study and sample collection. (b,c) Heatmaps reporting the relative quantity of plasmatic microRNAs (miRNAs) profiled in the two groups of subjects (linear fold changes in T1D relative to CTR) (**b**) and peripheral blood circulating immune cells enumerated in the two groups of subjects and normalized by row (linear fold changes in T1D relative to CTR) (**c**). Maximum blue = 0.5 and maximum red = 1.5 linear fold change compared to CTR. Actual numbers are reported in Supplementary Table S1.

For the present study, T1D children at diagnosis (*n* = 88) and sex-age matched healthy controls (CTR, *n* = 47) were recruited (Table 1). In peripheral blood, we assessed the immunophenotype by quantifying 18 circulating immune cell populations and profiled 60 plasmatic miRNAs by RT-qPCR (Figure 1 and Methods). Several differences in enumeration of immune cell populations and quantification of plasmatic miRNAs were observed between the CTR and T1D subjects (Figure 1b,c and Supplementary Table S1). Since T1D patients showed a significantly lower body mass index (BMI) than the healthy control group (Table 1), we performed logistic regression models adjusted for BMI. A mild but significant peripheral leukopenia paralleled by a reduced number of NK cells was found to be associated with T1D at diagnosis. Regarding miRNAs, our results suggest that an increase in four members of the let-7 family (let-7c-5p, let-7d-5p, let-7f-5p, and let-7i-5p) as well as miR-146a-5p and miR-423 (both the -3p and -5p molecules) and an appreciable decrease in miR-140-3p and miR-143-3p may be associated with T1D and not confounded by the BMI effect (Table 2). In particular, miR-140-3p was found to be significantly decreased by T1D disease upon correction for multiple testing. Receiver-operating characteristic (ROC) curves were then evaluated to determine the discriminatory performances of these T1D-associated parameters. Notably, the ROC curves showed areas under the curve (AUC) consistently above 0.7 for the identified plasmatic miRNAs, suggesting a better performance of miRNAs, compared to immune cells, in discriminating T1D and healthy children (Table 2).

### 2.2. Significant Change in Plasmatic miRNAs and Peripheral Blood Circulating Immune Cells is Associated with Different Capability of Insulin Secretion in T1D Children at Diagnosis

**Figure 2:**
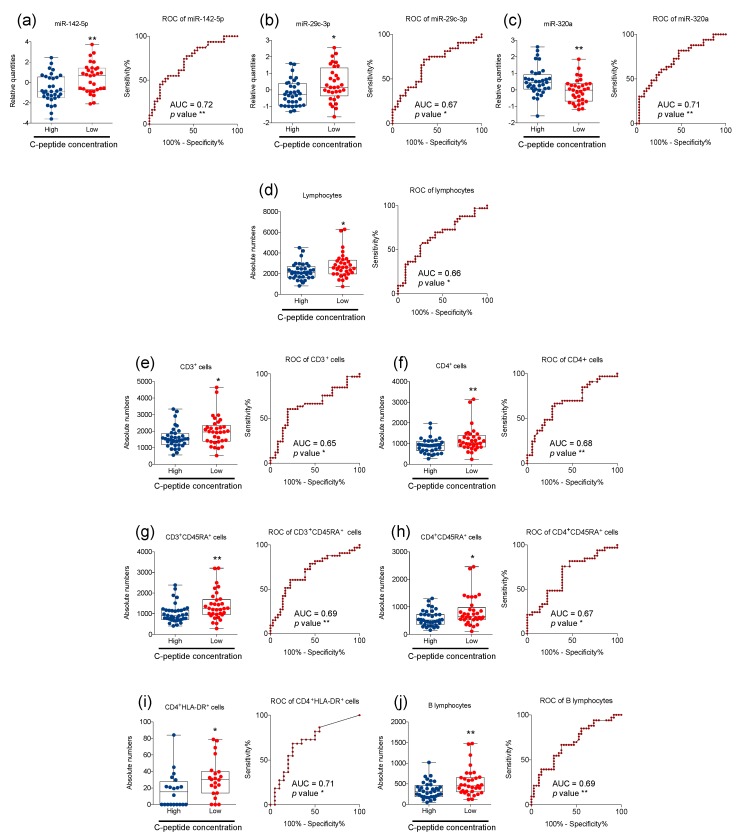
Association of miRNAs and immune cells with residual insulin secretion in T1D children at diagnosis. Whisker plots showing the relative quantities (expressed as log2) of plasmatic miRNAs (a–c) and the absolute number/mm^3^ of volume for the indicated peripheral blood circulating immune cells (d–j) in recent onset T1D patients with different levels of serum C-peptide, as indicated (high ≥ 0.4 ng/ml; low < 0.4 ng/mL). For each parameter, receiver-operating characteristic (ROC) curves showing the capability to discriminate between different groups of T1D patients, areas under the curve (AUC), and *p* values are also reported (* *p* < 0.05, ** *p* < 0.01). For direct comparisons, Mann-Whitney tests were performed: * p < 0.05, ** *p* < 0.01. Number of children with high serum C-peptide = 33 and low serum C-peptide = 31 for (**a**); 34 and 32 for (**b**); 36 and 33 for (**c**–**h**,**j**); 20 and 22 for (**i**).

T1D subjects in our cohort showed different residual β cell mass as testified by fasting C-peptide levels which ranged from 0.1 to 1.9 ng/mL (0.50% ± 0.39% mean ± SD; Table 1). Thus, T1D subjects were dichotomized into two different groups, namely, T1D subjects with higher (≥0.4 ng/mL) and lower (<0.4 ng/mL) residual C-peptide levels. Next, we evaluated whether plasmatic miRNAs and/or peripheral immune cells were associated with different insulin secretion capabilities. We found that miR-142-5p and miR-29c-3p were higher in T1D with low C-peptide levels, while miR-320a was significantly reduced in these subjects (Figure 2a–c). Furthermore, children with a reduced serum C-peptide concentration displayed higher absolute numbers of peripheral blood circulating total lymphocytes, CD3^+^, CD4^+^, CD3^+^CD45RA^+^, CD4^+^CD45RA^+^, CD4^+^HLA-DR^+^, and B cell subsets (Figure 2d–j). ROC measurement revealed that these differential parameters (both plasmatic miRNA and peripheral blood circulating immune cells) had a high degree of discrimination for T1D subjects with different residual insulin secretion (Figure 2). Of note is that plasmatic levels of miR-142-5p showed the best performance for classifying these sub-groups of T1D patients (Figure 2a).

### 2.3. Correlation of Plasmatic miRNAs with Glycemic Control in Children with T1D at Diagnosis

**Figure 3:**
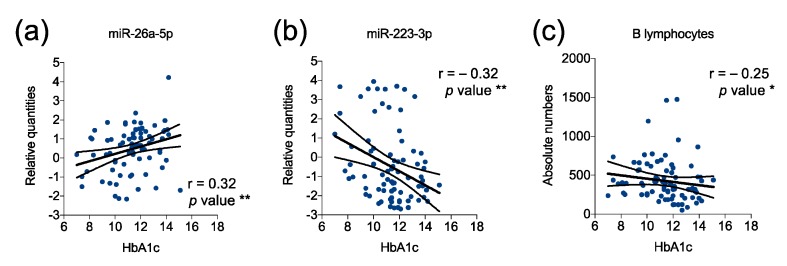
Statistically significant correlations of miRNAs and immune cells with Hb1Ac in children with T1D at diagnosis. Scatter plots showing the one-to-one correlations between relative quantities of the indicated plasmatic miRNAs (**a**,**b**) and absolute number/mm^3^ of volume for peripheral blood circulating B lymphocytes (**c**) with the percentage of glycated hemoglobin (HbA1c) in T1D children at diagnosis. Spearman r values and *p* values are also reported. * *p* < 0.05, ** *p* < 0.01. Numbers of children = 80 (**a**), 81 (**b**), and 88 (**c**).

Hemoglobin A1c (HbA1c) reflects a 90-day moving average of blood glucose concentrations and its elevated levels have been proposed as an alternative criterion for the diagnosis of diabetes [42]. HbA1c levels in T1D children at diagnosis (whose miRNA levels were analyzed with qPCR profiling) varied from 7.0% to 15.1% (11.34% ± 1.79% mean ± SD; Table 1). Of 60 profiled plasmatic miRNAs we found two miRNAs (3.3%) to be significantly correlated with HbA1c levels. To be more detailed, miR-26a-5p showed a positive correlation and miR-223-3p an inverse correlation with HbA1c levels (Figure 3a,b). Although these associations are weak in their nature (r = 0.3), they are stronger than the only statistically significant association found among peripheral blood circulating immune cells analyzed, namely that between the absolute number of B lymphocytes and the percentage of HbA1c (Figure 3c).

### 2.4. Plasmatic miR-29c-3p Is Significantly Associated with Diabetic Ketoacidosis in T1D Children at Diagnosis

**Figure 4:**
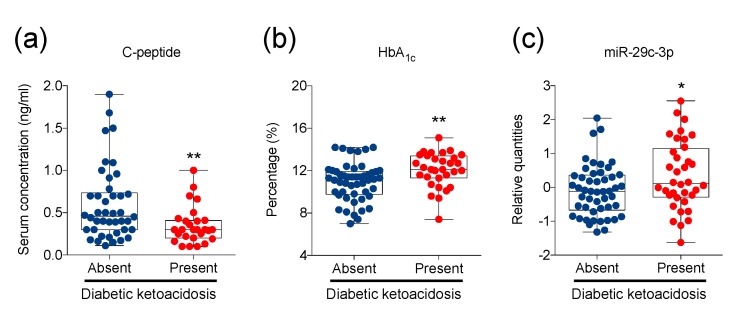
Significantly higher levels of plasmatic miR-29c-3p in the presence of diabetic ketoacidosis (DKA). (**a**,**b**) Whisker plots showing serum concentration of C-peptide (**a**), percentage of HbA1c (**b**), and relative quantities of plasmatic miR-29c-3p (**c**) in recent onset T1D children who presented or did not present DKA, as indicated. Mann-Whitney tests were performed: * *p* < 0.05, ** *p* < 0.01. Numbers of children with no DKA = 42 (**a**), 50 (**b**), and 49 (**c**). Numbers of children with DKA = 27 (**a**), 31 (**b**), and 36 (**c**).

Thirty-five out of 88 (39.8%) T1D children at diagnosis presented DKA, which is in line with other reports [5,9,10]. In accordance with previous observational studies [11,12], our data show that T1D children who presented with DKA also showed a higher degree of pancreatic function loss and higher levels of glycated hemoglobin (Figure 4a,b). Our results reveal a significant difference in total enumeration of peripheral blood circulating leukocytes, but no specific tested lymphocytic sub-population was found to associate with DKA (not shown). Comparison of plasmatic miRNA quantitative data between subjects with and without DKA instead shows statistically significant differences for miR-29c-3p; specifically, relative quantities of plasmatic miR-29c-3p were higher in T1D subjects with DKA at diagnosis (Figure 4c). In order to evaluate the potential biological effect of the plasmatic slight miR-29c-3p increase in T1D children with ketoacidosis, we focused on the list of genes experimentally validated as direct targets of this miRNA, as reported in the miRTarBase database (number of target mRNAs demonstrating strong experimental evidence = 66, http://miRTarBase.mbc.nctu.edu.tw/, release 7.0). String analysis then highlighted that these 66 genes are functionally linked significantly more than expected (275 edges, protein–protein interaction enrichment *p* value < 1.0 × 10^−16^). When analyzing the biological process enrichment in the functional network, the most enriched process that emerged was “*extracellular matrix organization*” (GO:0030198) (Supplementary Figure S1).

### 2.5. Correlation Analysis Reveals a T1D-Specific Interconnection between Plasmatic miRNAs and Peripheral Blood Circulating Immune Cells

**Figure 5:**
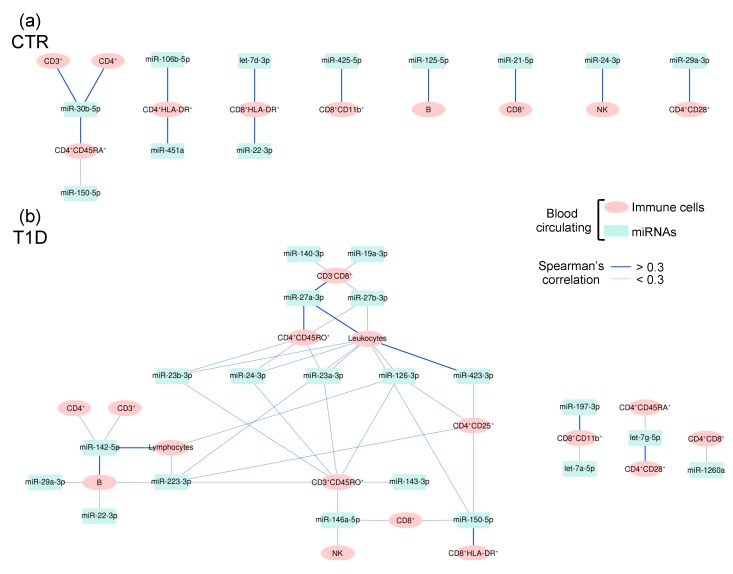
Correlation network between miRNAs and immune cells in healthy and T1D subjects. A positive correlation between the relative quantities of plasmatic miRNAs (green) and the absolute number/mm^3^ of volume for peripheral blood circulating immune cells (pink) in CTR (**a**) and T1D children at diagnosis (**b**) is expressed by blue edges. Edge thickness expresses the level of Spearman’s correlation, r, as indicated. Neither miRNA–miRNA nor cell–cell correlations are reported.

To evaluate a possible interconnection between the dysregulation of plasmatic miRNAs and the frequency of peripheral blood circulating immune cells in T1D, we analyzed the relationship between miRNAs and immune cells in both CTR and T1D children’s plasma. Under healthy conditions, significant connections between plasmatic miRNAs and peripheral blood circulating immune cells were found to be scant and mostly composed of a single edge relation (Figure 5a and Supplementary Table S2). In particular, the positive correlation between the absolute number of peripheral blood circulating CD4^+^CD45RA^+^ cells and plasmatic relative quantity of miR-150-5p stood out, since an increase in the blood of this same miRNA has been previously shown to mark lymphocyte activation in healthy children [43]. Significant connections between plasmatic miRNAs and peripheral blood circulating immune cells dramatically increased and also changed under diabetic compared to healthy conditions. For example, miR-150-5p was significantly correlated with activated CD8^+^HLA-DR^+^ cells in T1D but not in healthy children (Figure 5b and Supplementary Table S3). The specific profile (quantity and quality) of these connections in T1D children suggests a disease-specific miRNA association with diabetic immune dysregulation. Among others, miR-146a-5p (significantly associated with T1D onset, Table 2) was positively correlated with both NK and CD3^+^CD45RO^+^ cell absolute numbers, and miR-423-3p (also significantly associated with T1D onset) was correlated with peripheral blood circulating CD4^+^CD25^+^ (Figure 5b). MiR-142-5p was found to be strongly correlated with B lymphocytes, but also, more moderately, with CD3^+^, CD4^+^, and total lymphocyte cell numbers; all blood circulating cell populations were found to be augmented, together with this miRNA, in the plasma of T1D children with a lower serum concentration of C-peptide compared to children with a higher serum concentration of C-peptide (Figure 2). Plasmatic miR-223-3p, which was significantly associated with glycated hemoglobin (Figure 3b), represented a correlation hub: it correlated with total leukocyte, total lymphocyte, B, CD3^+^CD45RO^+^, and CD4^+^CD25^+^ cell absolute numbers (Figure 5b and Supplementary Table S3). On the other hand, miR-29c-3p, associated with both C-peptide secretion level and DKA, did not correlate with any of the enumerated blood circulating lymphocytic populations.

## 3. Discussion

T1D is a complex, multi-factorial disease driven by a combination of genetic makeup and a plethora of potential environmental triggers. As a result, age at diagnosis as well as immune cell alterations can consistently vary among subjects with T1D. In particular, the plasticity of the immune system implies a variability in the responses which is reflected in the involvement of different cell types and cytokines driving autoimmunity. This aspect, coupled with the different ability of β cells to cope with ongoing autoimmune destruction, results in different rates of progression to permanent hyperglycemia among patients [1,2,3,4,5,6]. Furthermore, the traditional idea that pancreatic damage is a mere consequence of autoimmune progression has been shaken by evidence for a whole-pancreas disease, characterized by throughout structural and functional alterations as well as immune cell infiltration already present in early disease stages. Extensive fibrosis, arteriosclerosis, acinar atrophy, and leucocytic infiltration are common histological abnormalities found in T1D subjects, suggesting that widespread pancreatic inflammation caused by a non-antigen specific pathogenesis may importantly concur to β cell destruction by the immune system [44,45]. Questions certainly remain concerning the pathogenetic implications of cell–cell communication within the pancreatic islets as well as the regeneration/proliferation and apoptosis of the β cells for T1D [46,47].

However, very little has been described regarding the infiltrating cell phenotype and accumulation, antigen specificity, and motility/circulation among the pancreas, lymph nodes, and blood. Whether pancreatic tissue residency and transit of immune cells are directly required for disease pathogenesis or rather constitute a bystander signature of inflammation remains undisclosed. On the other hand, we now know that immune cells (both tissue resident and peripheral blood circulating) secrete large amounts of miRNAs by active and passive mechanisms, which is linked to different functional states or damage [43,48,49,50]. The modulation of these miRNAs, released in the extracellular space and into peripheral blood, actually depends on the presence of functionally active lymphocytes in vivo and indeed may be registered in subjects affected with auto-immunity, representing ideal candidate mediators and biomarkers for complex multi-factorial diseases like T1D [43,48,49,50].

To our knowledge, this study is the first to test the association between peripheral blood co-circulating miRNAs and immune cells in the context of T1D disease onset and severity. The results of the study indicate that in T1D children plasmatic miRNA dysregulation and peripheral blood circulating T and B lymphocyte subset imbalance may indeed be related, since these parameters were found to be more frequently correlated in comparison with healthy conditions (Figure 5). In addition, the results also suggest that specific plasmatic miRNAs outperform peripheral blood immune cell counts in identifying T1D status, residual pancreatic function, and presence of DKA (Table 2, Figure 2 and Figure 4).

Given the observational nature of our study, we cannot infer whether the identified associations are causally linked to T1D pathogenesis. Nevertheless, all miRNAs found to be dysregulated in this study have been previously linked to T1D as either biomarkers or potentially involved in its pathogenesis. In particular, of the seven plasmatic miRNAs that we found robustly associated with T1D disease onset (Table 2), four belong to the let-7 family (let-7c, -7d, -7f, -7i), a central regulator of mammalian glucose metabolism. Global and pancreas-specific overexpression of let-7 results in impaired glucose tolerance, while let-7 inhibition leads to an insulin-sensitized state that can resist high fat diet-induced diabetes, and it is sufficient in this case to treat glucose tolerance impairment [51,52]. Moreover, let-7 family members regulate important aspects of immune cell biology, affecting lymphocyte cell determination, activation, effector function, and anergy, and extracellular let-7 miRNAs exert a role in the suppressive action and differentiation potential of CD4^+^ T regulatory cells [53,54,55,56,57,58].

The association of mir-26a-5p and 223-3p with HbA1c, coupled by the lack of an ability of these two miRNAs to discriminate between T1D patients and controls, suggest that they are altered as a consequence of chronic hyperglycemia. In this respect, miRNAs associated with immune alterations and parameters of pancreatic function, rather than with glycemic status, may represent better candidates for future studies regarding both markers and mediators of T1D disease.

Here, we found that C-peptide levels were inversely correlated with frequencies of total lymphocytes, B lymphocytes (with an essential role in adaptive immunity because of antibody production and antigen presentation), CD3^+^ and CD4^+^ T lymphocytes, and activated (HLA-DR^+^) CD4^+^ T cells, indicating that changes in these cell populations might be associated with more extensive destruction of β cells. Among the miRNAs also associated with serum C-peptide concentration, we identified plasmatic miR-29c-3p, which was univocally up-regulated in children who manifest DKA at T1D diagnosis (Figure 4). Several studies in mice and rats have associated miR-29c-3p dysregulation with impaired glucose transporter expression and glycemic control in skeletal muscle, exacerbated vascular remodeling, and hepatic negative regulation of insulin signaling in diabetes [59,60,61,62]. Less information is available on the involvement of miR-29c-3p in human diabetes. The capability of miR-29c-3p to trace a more severe manifestation of T1D may be very relevant to predicting higher risks of long-term diabetic complications, as DKA at diagnosis is associated with poor long-term glycemic control [11,12]. The significant enrichment in genes involved in extracellular matrix (ECM) organization for the list of miR-29c-3p-validated mRNA targets may functionally link miRNA-increased plasmatic levels in more severe T1D patients with data, indicating that a damaged ECM in the islets concurs with the breach of β cell homeostasis and loss of physical and immunoregulatory barriers against immune cell infiltration [63,64]. This intriguing hypothesis will deserve further exploration in the future.

In conclusion, we have here unveiled a blood co-circulating miRNA/immune cell subset combined modulation, which, upon proper validation in further studies, may mirror insulin secretion capability, glycemic control, and DKA in T1D children at diagnosis. Our observation that plasmatic miRNAs sense pathologically relevant immune cell dynamics more efficiently than peripheral blood circulating immune cells and better reflect the pancreas-specific etiopathogenetic progression may improve T1D patients’ stratification, thus supporting relevant aspects of clinical management.

## 4. Materials and Methods

### 4.1. Subjects

Children who received the diagnosis of type 1 diabetes (*n* = 88) were recruited at the Department of Translational Medical Science, Pediatrics (University of Naples Federico II, Naples Italy) after glycemic stabilization on exogenous insulin achieved in 10 days, and all of those recruited were positive for at least two anti-islet autoantibodies. Diabetes was defined according to the Global International Diabetes Federation/International Society for Pediatric and Adolescent Diabetes Guidelines for Diabetes in Childhood and Adolescence [65] and included symptoms of diabetes in addition to casual plasma glucose concentration ≥11.1 mmol/L (200 mg/dL) or fasting plasma glucose ≥7.0 mmol/L (≥126 mg/dL) or 2 h postload glucose ≥11.1 mmol/L (≥200 mg/dL) during an oral glucose tolerance test, and HbA1c ≥6.5%. The healthy controls (*n* = 47) used in the study were recruited at the Department of Translational Medical Science, Pediatrics (University of Naples Federico II) and selected using the following criteria: fasting blood glucose <5.5 mmol/L (<100 mg/dL), negative personal and familial history of autoimmune disorders, and negativity for islet autoantibodies at the 99^th^ percentile. Healthy subjects were matched for sex, age, and BMI with T1D subjects. T1D and healthy subjects with recent vaccinations or infections were excluded from the study.

### 4.2. Laboratory Testing

For T1D and healthy individuals a 6 mL blood sample was withdrawn at 8:00 a.m. into heparinized BD Vacutainers and processed within the following 4 h. An aliquot from each blood sample was used to perform immune cell profiling by flow cytometry and the remaining part of the sample was used for serum-based assays, which were performed at the Pediatrics department. Sera were centrifuged and kept at −80 °C until use. Fasting C-peptide levels were measured in duplicate serum samples at the same time for all samples using a commercial ELISA kit (Millipore Corporation, Bedford, MA, USA). Glucose levels were measured using the enzymatic hexokinase method and HbA1c was measured using high-performance liquid chromatography (HLC-723 G7, TOSOH Bioscience, Tokyo, Japan). Islet autoantibodies (GADA, IA-2A, IAA, and ZnT8) were measured using a commercial ELISA kit (Pantec, Turin, Italy).

### 4.3. Immunophenotypic Analysis

Additional blood samples were collected in heparin-tubes and were used for immune cell profiling (see Figure 1). Whole blood cells were analyzed with a clinical-grade hematocytometer to determine absolute lymphocyte numbers in each sample. One-hundred microliters of blood were incubated 30 min at room temperature with the specific antibody combinations. Red blood cells were lysed using BD FACS lysing Solution 2 (BD Bioscience, Franklin Lakes, New Jersey, USA) for 10 min and samples were subsequently washed and resuspended in 300 μL phosphate-buffered saline (PBS). Flow cytometry was carried out on cells gated on CD45^+^—Side Scatter (SSC). Immunophenotypic analysis was performed with an EPICS XL flow-cytometer (Beckman Coulter, Brea, CA, USA) using the Beckman Coulter software program XL System II. Triple combinations of different human mAbs (FITC- and phycoerythrin (PE)-anti-CD3, PE- and PE-cyanine (PC) 5-anti-CD4, PC5-anti-CD8, PE-anti-CD16, PC5-anti-CD19, PE-anti-CD25, FITC-anti-CD45, PE-anti-CD56, PE anti-CD45RA, PE anti-CD28, PE anti-HLA-DR, and PE anti-CD11b), all from Coulter Immunotech (Marseille, France), were used to identify different cell populations. The gating strategy is reported in Supplementary Figure S2. Additional plasma samples from the same subjects analyzed for the immunophenotype were used for miRNA studies.

### 4.4. Plasmatic miRNA Quantification

After thawing, the samples were centrifuged at 3000 × *g* for 5 min at 4 °C to remove cryoprecipitates and cell debris. Sample hemolysis was measured at 414 nm. Total RNA was isolated from 300 μL human plasma in the presence of MS2 carrier RNA (Roche, Basel, Switzerland) using the miRCURY RNA Isolation Kit—Biofluid (Qiagen, Hilden, Germany) according to the manufacturer’s instructions. RNA samples were heparinase-treated for 1 h at 25 °C. During the first phase of the study we profiled 179 miRNAs known to be detectable in human peripheral blood (Exiqon/QIAGEN, Serum/Plasma miRNA PCR Panels) in type 1 diabetes patients at recent onset (*n* = 57) and healthy controls (*n* = 26). This first study phase was not intended for the carrying out of differential expression analysis but instead to select for a reduced list of miRNAs with the most robust expression level in plasma of the children of our cohort. We thus selected 60 miRNAs based on the percentage of detection (>90% of analyzed samples) and average expression value (Ct < 35). In the second study phase, we quantified only this subset of 60 miRNAs in additional plasma samples of type 1 diabetes patients at recent onset and healthy controls (*n* = 31 and *n* = 21, respectively), using miRCURY LNA™ miRNA Custom PCR Panels (Qiagen) according to the manufacturer’s instruction. For a list of the selected miRNAs, refer to Figure 1. RNA was reverse-transcribed with a miRCURY LNA™ Universal RT Kit (Qiagen). Quantitative real-time PCR was performed using a SYBR Green master mix (Qiagen) with LNA™-based miRNA primers (Qiagen). Plates were run on an ABI QuantStudio 6K Flex (the same cycling conditions and parameters were maintained throughout the study). Four wells on each plate were assigned for inter-plate calibration, eliminating run-to-run variation when comparing multiple plates. Furthermore, in order to monitor for RNA isolation efficiency and retro-transcription reproducibility, we routinely spiked-in synthetic RNA resembling miRNAs (i.e., UniSp2 and UniSp4 to lysis buffer and *Caenorhabditis elegans* miR-39 (cel-miR-39-3p) to cDNA synthesis), which, by being ultimately measured by RT-qPCR, provided an internal reference. Ct values above 37 were considered undetectable. Relative values were calculated with the ΔCT method, using the miRNA global mean as a biological normalization factor, and miRNA quantitative data were graphed as log2 fold change to the relatively healthy control cohort. 

### 4.5. MiRNA Target Gene Analysis

Validated mRNA gene targets for specific miRNAs (miR-29c-3p) were identified using miRTarBase Release 7.0 (mirtarbase.mbc.nctu.edu) [66]. STRING (https://string-db.org) analysis was then performed on validated gene targets to visualize the protein–protein interaction network, and enriched biological pathways were recognized using the KEGG gene database.

### 4.6. Statistics

Distribution of continuous variables was tested for normality using the Kolmogorov-Smirnov test. Comparisons between groups were performed by either an unpaired Student’s *t*-test or a non-parametric Wilcoxon test (in the case of two groups) and by either ANOVA or Kruskal-Wallis (in the case of three or more groups), respectively, for normally and not normally distributed variables. Whisker plots were used to represent distributions of absolute numbers of peripheral blood circulating immune cells and fold changes in plasmatic miRNA expression. Correlations between miRNAs and peripheral blood immune cells were graphically represented by correlation networks. To evaluate the association between either subject status (controls versus T1D) or T1D severity (C-peptide secretion, glycated hemoglobin, and presence of DKA) and collected variables, logistic regression models were implemented. ROC curves were drawn for models and the corresponding areas under the curve were calculated. Statistical analyses were performed using SAS software (version 9.4) and statistical significance was defined as *p* value < 0.05. Graphical representations were drawn using GraphPad Prism software 7, with data presented as the mean ± SEM, unless stated otherwise, and with R software (R version 3.4.4) by using the corrplot library (http://cran.r-project.org/).

### 4.7. Study Approval

The study was approved by the Institutional Review Board (IRB) of the University of Naples “Federico II”, prot. no. 200/16, and parents gave informed consent. We complied with all relevant ethical regulations.

## Figures and Tables

**Table 1:** Baseline characteristics of type 1 diabetes (T1D) children at diagnosis and healthy controls (CTR) recruited for the study. Comparisons were performed using either Student’s *t*-test or a Chi-squared test (§). Legend: n.s., not significant; n.a., not available. * *p* < 0.05.

Baseline Characteristics	CTR	T1D	*p* value
Numbers of subjects	47	88	-
Age (years)	8.44 ± 3.25	8.97 ± 3.68	n.s.
Gender (%M)	45.65	54.55	n.s.(§)
Body mass index (kg/m^2^)	19.72 ± 4.82	17.43 ± 3.44	*
C-peptide (ng/mL)	n.a.	0.50 ± 0.39	-
Ketoacidosis at diagnosis (yes/no/n.a.)	n.a.	35/52/1	-
Glycated hemoglobin (%)	n.a.	11.34 ± 1.79	-
Insulin dose (IU/kg/day)	n.a.	0.65 ± 0.30	-
Other autoimmune disorders (yes/no)	0/47	15/73	*(§)

**Table 2:** Association of miRNAs and immune cells with T1D disease status at diagnosis. Multivariate logistic models, adjusted for body mass index (BMI), were evaluated for plasmatic miRNAs, expressed as log2 fold change in T1D compared to CTR children, and peripheral blood circulating immune cells, expressed as absolute number/mm^3^ of blood. Legend: NK, natural killer; OR, odds ratio; CI, confidence interval.

Variable	CTR (Mean ± SD)	T1D (Mean ± SD)	Adj. OR (95% CI) for One Unit of Parameter Increase	*p* Value	Adjusted for	AUC
let-7c-5p*	0 ± 0.73	0.5 ± 0.91	2.81 (1.37–5.74)	0.0046	BMI	0.769
let-7d-5p*	0 ± 0.83	0.44 ± 0.9	2.49 (1.19–5.19)	0.0150	BMI	0.732
let-7f-5p*	0 ± 0.85	0.81 ± 0.88	4.15 (1.82–9.48)	0.0007	BMI	0.845
let-7i-5p*	0 ± 0.94	0.41 ± 0.61	3.05 (1.56–5.98)	0.0012	BMI*	0.770
miR-140-3p*	0 ± 1	−0.57 ± 0.93	0.36 (0.22–0.62)	0.0001	BMI*	0.793
miR-143-3p*	0 ± 0.8	−0.74 ± 0.86	0.41 (0.20–0.85)	0.0170	BMI	0.700
miR-146a-5p*	0 ± 0.94	0.41 ± 0.84	2.51 (1.47–4.28)	0.0008	BMI*	0.742
miR-423-3p*	0 ± 0.72	0.46 ± 0.83	2.27 (1.15–4.47)	0.0181	BMI	0.729
miR-423-5p*	0 ± 1.1	0.56 ± 1.01	2.24 (1.38–3.65)	0.0011	BMI*	0.763
Leukocytes	6472.79 ± 2624.42	5233.22 ± 1818.89	0.75 (0.61–0.93) °	0.0080	BMI	0.698
NK	286.39 ± 210.22	179.23 ± 108.48	0.68 (0.49–0.93) §	0.0155	BMI*	0.693

* Significant adjusting variable. ° For 1000 unit increase. § For 100 unit increase.

## Supplementary Materials

Supplementary materials can be found at https://www.mdpi.com/1422-0067/21/2/477/s1.

## Abbreviations

miRNA	microRNAs
LNA	locked nucleic acid
cel-miR-39-3p	Caenorhabditis elegans miR-39
ROC	receiver operating characteristics
