# Supplementary material for: Blood Co-Circulating Extracellular microRNAs and Immune Cell Subsets Associate with Type 1 Diabetes Severity
Source: Int J Mol Sci. 2020 Jan 11;21(2):477. doi: 10.3390/ijms21020477 (PMC7013659; doi:10.3390/ijms21020477)
Supplement: Supplementary file 1 [file ijms-21-00477-s001.pdf]

International Journal of *Molecular Sciences*

MDPI

**Variable (miRNAs)**	**CTR**	**T1D**
hsa-let-7a-5p	0±0.79	0.32±0.99
hsa-let-7b-5p	0±0.91	0.01±0.94
hsa-let-7c-5p	0±0.73	0.5±0.91
hsa-let-7d-3p	0±0.84	0.15±0.84
hsa-let-7d-5p	0±0.83	0.44±0.9
hsa-let-7f-5p	0±0.85	0.81±0.88
hsa-let-7g-5p	0±0.82	0.24±0.85
hsa-let-7i-5p	0±0.94	0.41±0.61
hsa-miR-101-3p	0±0.85	0.1±0.74
hsa-miR-103a-3p	0±0.88	0.13±0.94
hsa-miR-106a-5p	0±0.9	0.23±0.73
hsa-miR-106b-5p	0±0.96	0.23±0.75
hsa-miR-125-5p	0±1.01	0.02±1.37
hsa-miR-126-3p	0±1.36	0.01±1.41
hsa-miR-1260a	0±1.32	-0.39±1.7
hsa-miR-140-3p	0±1	-0.57±0.93
hsa-miR-142-3p	0±0.81	-0.11±0.93
hsa-miR-142-5p	0±1.35	-0.17±1.41
hsa-miR-143-3p	0±0.8	-0.74±0.86
hsa-miR-144-3p	0±1.08	0.34±1.35
hsa-miR-145-5p	0±0.77	-0.44±1.08
hsa-miR-146a-5p	0±0.94	0.41±0.84
hsa-miR-150-5p	0±0.87	0±0.78
hsa-miR-15a-5p	0±0.95	0.29±0.87
hsa-miR-16-5p	0±1.16	-0.24±0.87
hsa-miR-181a	0±0.75	-0.03±0.97
hsa-miR-185-5p	0±1.11	0.15±0.93
hsa-miR-192-5p	0±1.07	0.16±1.37
hsa-miR-197-3p	0±0.96	-0.49±0.69
hsa-miR-19a-3p	0±1.02	-0.18±1.01
hsa-miR-20a-5p	0±0.94	0.28±0.72
hsa-miR-21-5p	0±0.77	0.25±0.76
hsa-miR-22-3p	0±0.91	-0.09±1.24
hsa-miR-22-5p	0±4.31	0.25±3.58
hsa-miR-221-3p	0±1.16	0.2±1.1
hsa-miR-223-3p	0±2.35	-0.41±2.05
hsa-miR-23a-3p	0±0.95	-0.04±0.94
hsa-miR-23b-3p	0±0.83	-0.12±0.92
hsa-miR-24-3p	0±0.93	-0.04±0.9
hsa-miR-25-3p	0±0.82	-0.09±0.89
hsa-miR-26a-5p	0±1.22	0.39±1.25
hsa-miR-27a-3p	0±0.9	-0.05±1.3
hsa-miR-27b-3p	0±1.03	0.1±1.2
hsa-miR-29a-3p	0±1.1	-0.06±1.02
hsa-miR-29c-3p	0±0.85	0.09±0.9
hsa-miR-30b-5p	0±0.8	-0.16±0.81
hsa-miR-30c-5p	0±0.81	-0.11±0.83
hsa-miR-30d-5p	0±0.68	-0.16±0.75
hsa-miR-30e-5p	0±0.98	-0.42±0.83
hsa-miR-320a	0±0.99	0.29±0.8
hsa-miR-320b	0±0.9	0.46±0.99
hsa-miR-342-3p	0±0.87	-0.1±0.77
hsa-miR-361-5p	0±0.89	-0.16±0.92
hsa-miR-423-3p	0±0.72	0.46±0.83
hsa-miR-423-5p	0±1.1	0.56±1.01
hsa-miR-425-5p	0±0.56	-0.42±0.9
hsa-miR-451a	0±1.25	-0.02±1.22
hsa-miR-486-5p	0±1.43	0.08±1.15
hsa-miR-92a-3p	0±1	0.38±0.74
hsa-miR-93-5p	0±0.96	0.16±0.85
**Variable (immune cells)**	**CTR**	**T1D**
Leukocytes	6472.79±2624.42	5233.22±1818.89
Lymphocytes	2594.69±1037.03	2477.41±960.66
CD3+	1835.46±732.07	1790.36±717.49
CD4+	1030.78±432.12	1043.27±462.54
CD8+	637.51±285.33	589.38±273.31
NK	286.39±210.22	179.23±108.48
B	418.56±260.53	436.61±282.39
CD4+CD8+	16.43±16.19	16.62±18.53
CD3+CD45RA+	1260.16±574.87	1204.54±551.21
CD3+CD45RO+	560.66±266.73	590.61±289.05
CD4+CD28+	942.08±471.53	980.65±442.57
CD4+HLA-DR+	25.79±27.5	25.27±24.52
CD4+CD25+	42.01±66.79	28.99±25.73
CD4+CD45RA+	696.07±349.06	708.48±411.25
CD4+CD45RO+	326.69±157.54	344.82±177.02
CD8+HLA-DR+	16.82±22.07	23.53±24.55
CD8+CD11b+	119.69±87.19	108.78±115.72
CD3-CD8+	91.06±88.99	70.94±65.71

Supplementary Table 1. ***Quantification of miRNAs and immune cells in healthy controls and type 1 diabetic children.*** The list reports the relative quantity of plasmatic miRNAs profiled in in healthy controls (CTR) and type 1 diabetic children (T1D) (linear fold changes in T1D relative to CTR) and the absolute numbers of peripheral blood circulating immune cells enumerated in 1 mm^3 of blood.

Variable	by Variable	Spearman ϱ	Prob>|ϱ|
let-7d-3p	CD8+HLA-DR+	0.59	0.00642
miR-106b-5p	CD4+HLA-DR+	0.51	0.00315
miR-125-5p	B	0.34	0.01807
miR-150-5p	CD4+CD45RA+	0.30	0.03310
miR-21-5p	CD8+	0.33	0.01939
miR-22-3p	CD8+HLA-DR+	0.41	0.02854
miR-24-3p	NK	0.32	0.02327
miR-29a-3p	CD4+CD28+	0.41	0.03820
miR-30b-5p	CD3+	0.33	0.02653
miR-30b-5p	CD4+	0.40	0.00710
miR-30b-5p	CD4+CD45RA+	0.39	0.00714
miR-425-5p	CD8+CD11b+	0.34	0.03560
miR-451a	CD4+HLA-DR+	0.34	0.04941

Supplementary Table 2. ***Correlation between miRNAs and immune cells in healthy controls.*** The one-to-one significant positive correlations between relative quantities of plasmatic miRNAs and absolute numbers of blood circulating immune cells are reported together with Spearman r and p values.

Variable	by Variable	Spearman ρ	Prob>|ρ|
let-7a-5p	CD8+CD11b+	0.23	0.03056
let-7g-5p	CD4+CD28+	0.30	0.03841
let-7g-5p	CD4+CD45RA+	0.22	0.04555
miR-126-3p	CD3+CD45RO+	0.27	0.01091
miR-126-3p	CD4+CD25+	0.27	0.01577
miR-126-3p	Leukocytes	0.23	0.03645
miR-126-3p	Lymphocytes	0.24	0.02646
miR-1260a	CD4+CD8+	0.21	0.04762
miR-140-3p	CD3-CD8+	0.29	0.00705
miR-142-5p	B	0.45	0.00002
miR-142-5p	CD3+	0.25	0.02434
miR-142-5p	CD4+	0.23	0.03992
miR-142-5p	Lymphocytes	0.32	0.00393
miR-143-3p	CD3+CD45RO+	0.27	0.02887
miR-146a-5p	CD3+CD45RO+	0.30	0.00554
miR-146a-5p	CD8+	0.22	0.04302
miR-146a-5p	NK	0.22	0.03753
miR-150-5p	CD4+CD25+	0.23	0.03707
miR-150-5p	CD8+	0.25	0.01938
miR-150-5p	CD8+HLA-DR+	0.30	0.03191
miR-150-5p	Leukocytes	0.30	0.00508
miR-197-3p	CD8+CD11b+	0.42	0.00014
miR-19a-3p	CD3-CD8+	0.24	0.02546
miR-22-3p	B	0.24	0.03008
miR-223-3p	B	0.25	0.01657
miR-223-3p	CD3+CD45RO+	0.25	0.02048
miR-223-3p	CD4+CD25+	0.26	0.02050
miR-223-3p	Leukocytes	0.28	0.00792
miR-223-3p	Lymphocytes	0.27	0.01015
miR-23a-3p	CD3+CD45RO+	0.24	0.02445
miR-23a-3p	CD4+CD45RA+	0.24	0.02582
miR-23a-3p	Leukocytes	0.23	0.02931
miR-23b-3p	CD3+CD45RO+	0.26	0.01487
miR-23b-3p	CD4+CD45RA+	0.27	0.01115
miR-23b-3p	Leukocytes	0.27	0.01131
miR-24-3p	CD3+CD45RO+	0.29	0.00594
miR-24-3p	CD4+CD45RA+	0.28	0.00756
miR-24-3p	Leukocytes	0.30	0.00517
miR-27a-3p	CD3-CD8+	0.33	0.00196
miR-27a-3p	CD4+CD45RA+	0.34	0.00107
miR-27a-3p	Leukocytes	0.31	0.00377
miR-27b-3p	CD3-CD8+	0.21	0.04606
miR-27b-3p	CD4+CD45RA+	0.28	0.00740
miR-27b-3p	Leukocytes	0.27	0.01182
miR-29a-3p	B	0.26	0.01669
miR-423-3p	CD4+CD25+	0.23	0.04741
miR-423-3p	Leukocytes	0.33	0.00246

Supplementary Table 3. ***Correlation between miRNAs and immune cells in type 1 diabetic children at onset.*** The one-to-one significant positive correlations between relative quantities of plasmatic miRNAs and absolute numbers of blood circulating immune cells are reported together with Spearman r and p values.

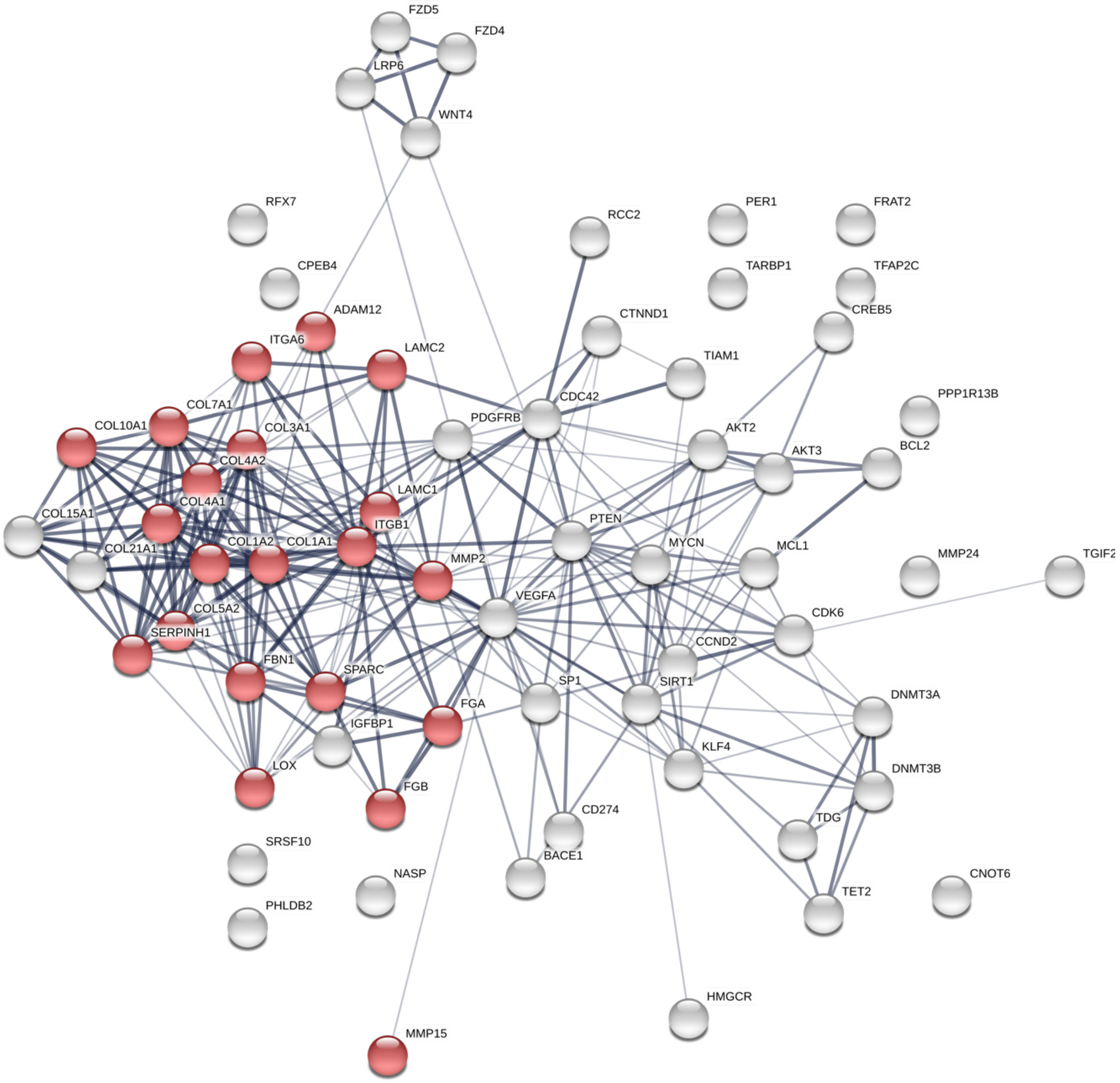

Supplementary Figure 1. ***Protein-Based Functional Connection of miR-29c-3p validated targets.***

STRING network analysis on miR-29c-3p validate targets. Network nodes (n=66) represent the protein product of the corresponding genes. Edges (n=275) represent protein-protein associations, i.e. joint contribution to a shared function, with no necessary physical interaction. Line thickness of network edges is based on confidence and indicates the strength of data support. Protein-protein interaction enrichment p-value<1.0e-16. In red, genes belonging to the biological process GO:0030198 (*extracellular matrix organization*). False discovery rate for the enrichment = 1.7e-18

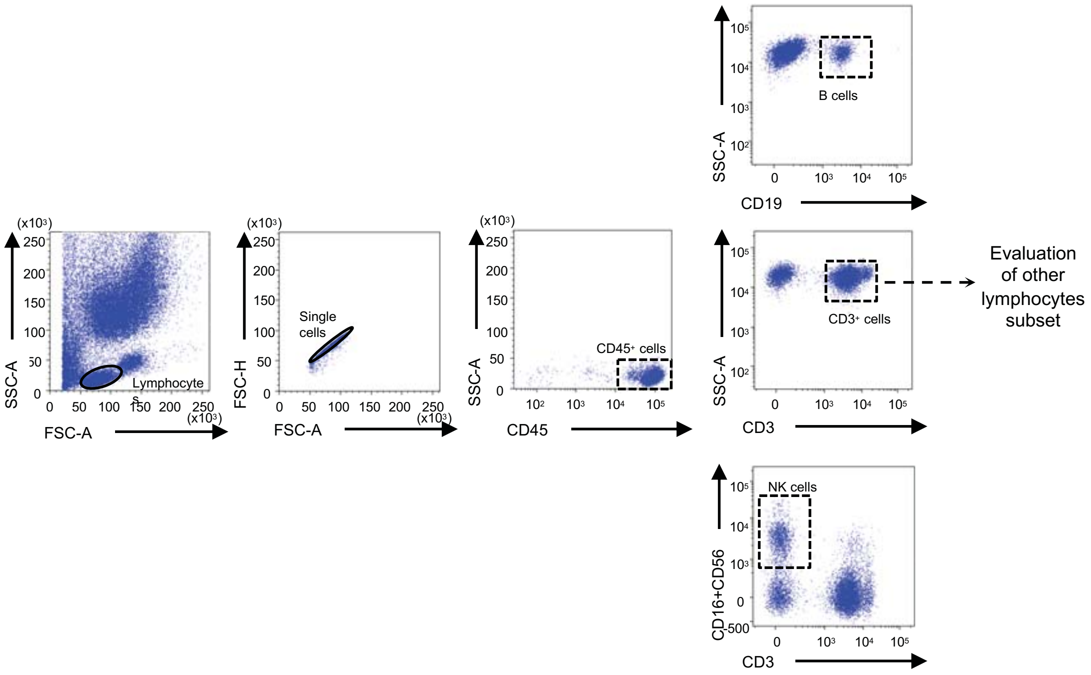

Supplementary Figure 2. ***Flow-cytometry gating strategy for the analysis of lymphocyte subsets.***

Representative flow-cytometry plots showing the gating strategy used to evaluate the frequency of immune cell subsets in PBMCs. Lymphocytes are gated based on SSC-A *versus* FSC-A; singlets are selected from the FSC-A *versus* FSC-H dot plot. Frequency of $CD3^+$ cells are identified based on CD3 expression; percentage of $CD19^+$ cells are evaluated for CD19 positive levels; NK cells are negative for CD3 marker and positive for the expression of CD16 and/or CD56 molecules. FSC-A, forward scatter area; SSC-A, side scatter area; FSC-H, forward scatter height.
